# Comparative Study of Digital Squamous Cell Carcinoma in Giant, Standard, and Miniature Schnauzers

**DOI:** 10.3390/ani13121990

**Published:** 2023-06-14

**Authors:** Heike Aupperle-Lellbach, Daniela Heidrich, David Conrad, Christoph Beitzinger, Nives Masala, Robert Klopfleisch, Tobias Müller

**Affiliations:** 1 LABOKLIN GmbH & Co. KG, 97688 Bad Kissingen, Germany; daniela.heidrich@freenet.de (D.H.); beitzinger@laboklin.com (C.B.); 2 Institute of Pathology, Department Comparative Experimental Pathology, School of Medicine, Technical University of Munich, 80333 Munich, Germany; 3 Anicura Aachen, Trierer Str. 652-658, 52078 Aachen, Germany; nives.masala@anicura.de; 4 Institute of Veterinary Pathology, Freie Universität Berlin, 14163 Berlin, Germany; robert.klopfleisch@fu-berlin.de; 5 Institute of Bioinformatics, University of Würzburg, 97070 Würzburg, Germany; tobias.mueller@uni-wuerzburg.de

**Keywords:** toe, dog, pathology, breed predisposition, survival time, multiplicity

## Abstract

**Simple Summary:**

In schnauzers, a breed predisposition to squamous cell carcinoma of the digit (dSCC) is well known. In terms of the breed, the variants of giant (GSs), standard (SSs), and miniature schnauzers (MSs), as well as the traits of body size and/or genetic background may have an impact on tumour occurrence and prognosis. We, therefore, conducted a retrospective analysis of the pathological findings of 478 digital SCCs from 417 schnauzers (227 GSs, 174 SSs, and 16 MSs). The results showed that MSs were older when digital SCC appeared, and lesions were mostly (90%) nodular. A gender predisposition for male GSs was identified (*p* < 0.05). In GSs, nodular lesions were larger than in MSs and SSs. In all breed variants, SCC was mostly diagnosed at the forelimbs, especially at digits 1, 2, and 5. In contrast, at the hindlimbs, the affected toes differed between GSs and SSs. Multiple dSCCs were more common in SSs than in GSs. If dSCC was the cause of death, the survival time was shorter than in dogs dying from other diseases. Metastases occurred in 20% of the cases and led to a significantly shorter survival time in GSs and SSs. The results showed various differences in the dSCC depending on the size variant of the schnauzer.

**Abstract:**

In schnauzers, a breed predisposition to squamous cell carcinoma of the digit (dSCC) is well known. The aim of this study was to compare the clinical and macroscopic findings of dSCCs in giant (GSs), standard (SSs), and miniature schnauzers (MSs). Methods: Pathology reports of 478 dSCCs from 417 schnauzers (227 GSs, 174 SSs, and 16 MSs) were retrospectively evaluated. Results: The MSs were older than the SSs and GSs (*p* ≤ 0.01). The male GSs were predisposed to dSCC (*p* < 0.05). In the GSs, the nodular dSCCs were larger than in the MSs (*p* ≤ 0.05) and SSs (*p* ≤ 0.001). The digital SCCs were mostly diagnosed at the forelimbs, especially at digits 1, 2, and 5. At the hindlimbs, the affected toes differed between the GSs and SSs. Multiple dSCCs were more common in SSs than in GSs (*p* = 0.003). If dSCC was the cause of death, the survival time was shorter than in dogs dying from other diseases (*p* = 0.004). Metastases occurred in 20% of the cases and led to a significantly shorter survival time in both the GSs and SSs (*p* < 0.001). Conclusions: The results showed various differences in the dSCC depending on the size variant of the schnauzer.

## 1. Introduction

The schnauzer is a dog breed with three size variants, namely the giant schnauzer (GS), standard schnauzer (SS), and miniature schnauzer (MS), and they have four main coat colours. GSs and SSs are black or pepper and salt while MSs appear as black, pepper and salt, and white or black-silver (Federation Cynologique International; FCI standard Nos. 181–183).

Squamous cell carcinoma (SCC) is a malignant neoplasm arising from the epidermal layer with varying degrees of keratinocyte differentiation [1]. The most common sites include the skin [2], the digit [3] and the oral cavity [4,5,6,7], with different biological behaviours. The clinical picture and diagnostic work-up of canine SCC at different anatomical sites have been described in detail [1].

When tumour samples from 1276 schnauzers (631 GSs, 378 SSs, and 267 MSs) were examined, significant differences in tumour incidence were detected between the giant, standard, and miniature schnauzers. For example, benign neoplasms were significantly more common in MSs than in GSs. Melanocytic neoplasms appeared more often in GSs than in MSs or SSs. Giant and standard schnauzers showed significantly fewer mammary tumours but more squamous cell carcinomas than MSs [8].

Regarding digits, SCC is the most commonly diagnosed tumour, accounting for 47.4% to 63.5%, and they mostly occur in animals between 6 and 13 years of age [9,10]. In GSs and SSs, as well as in black standard poodles, Rottweilers, and black Labrador retrievers, a breed predisposition to squamous cell carcinoma of the toe is well known [3,9,10,11,12,13,14,15]. The clinical and histopathological characterisation of 154 dogs with dSCC identified Beaucerons and Briards as the other overrepresented breeds [12]. In a recent study of 79 dogs with SCC of the digit (84.8% dark coated), schnauzers represented approximately one-third of the study population and had a poorer outcome compared with other breeds [3]. A previous study by our group confirmed that SCC is histomorphologically more aggressive in black schnauzers and other dark-coated dogs than in other canine breeds [16]. 

One of the most common pathogeneses underlying UV-related human cutaneous SCC are p53 gene (TP53) mutations, which alter the p53 pathways that regulate the cell cycle and maintain genomic stability [17]. Thus, outdoor animals, or those with a light coat (e.g., Dalmatians, boxers, bull terriers, beagles, and pointers), are prone to develop SCC of their body’s skin [2,18,19,20,21,22]. Other less common factors that have been suggested are papillomavirus, which has been detected in canine cutaneous SCC [22,23,24], and also in a history of trauma or burns [25]. An underlying genetic predisposition has been hypothesised because UV-related damage is unlikely in digital/subungual SCCs; additionally, black-haired dogs are predisposed to this disease, and multiple digits can be affected [12,26]. A copy number variation (CNV) at the KITLG gene is responsible for coat colour intensity [27]. Furthermore, the CNV at the KITLG gene is associated with the predisposition to digital SCC in black poodles [28] and black giant schnauzers [29], as well as with the degree of the digital SCC’s histological aggressiveness [30]. However, other genetic factors may play a role, suggesting that there are multiple pathways leading to the final oncogenesis of digital SCC [28].

As previously shown, among the schnauzer breed variants, body size and/or genetic background seem to have an impact on tumour occurrence and prognosis [3,8,26]. However, in the literature on digital SCC, there is only one study on 38 cases of digital SCC in schnauzers, which included 12 GSs (32%), 24 SSs (63%), and 2 MSs (5%) [31]. Most other publications made no distinction of the schnauzer size variants for specific clinical or pathological parameters [3,9,12,30]. Thus, the aim of the present study is to compare the clinical and macroscopic findings of digital SCC in giant, standard, and miniature schnauzers based on a high number of cases.

## 2. Materials and Methods

### 2.1. Data Curation

Cohort 1: Between 2013 and 2022, histopathological samples from 1836 GSs, 2113 SSs, and 1595 MSs were submitted to the pathology department of LABOKLIN GmbH & Co. KG, Bad Kissingen, Germany. As all samples were routine diagnostic submissions, there was no need to submit a request for animal testing or to obtain approval from the ethics committee. This approach was supported by a decision from the local government (RUF-55.2.2-2532-1-86-5). In total, 388 dSCCs deriving from 356 dogs (205 GSs, 135 SSs, and 16 MSs) were included in this study;

Cohort 2: In addition, 90 dSCC samples from 61 dogs (22 GSs and 39 SSs) submitted to other pathology laboratories were included. These had been part of another study with a focus on clinical data.

Thus, in total, 478 digital squamous cell carcinomas from 417 schnauzers (227 GSs, 174 SSs, and 16 MSs) were included in this retrospective study. 

Schnauzer-mixed breeds were excluded from this study. Only cases with given information about age and sex, as well as a diagnosis of digital squamous cell carcinoma were selected for further evaluation. 

The data collected the submission forms included breed, age, and sex. In several cases, the tumour site (affected leg and/or toe) was given. Further information about clinical findings, treatments, and outcomes were requested by telephone or email from the owners and/or the treating veterinarians as much as possible. In the end, information about the outcome was available in 55/417 cases. Unfortunately, in most cases, the slides were no longer available from the archives, and the histopathological findings could not be revalidated for this study.

### 2.2. Statistics

All statistical analyses were carried out with the statistical software R, version 4.2 [32]. Fisher’s exact test was used to test the association between the categorical variables. To test for a given proportion (e.g., forelimbs and hindlimbs being equal to 50% in a given breed), the proportion test was applied, as implemented in the R function “prop.test”. The median of the two groups was compared with the Wilcoxon rank sum test; for those of more than two groups, the Kruskal–Wallis test was utilized instead. Here, the non-parametric post hoc test of Nemenyi for multiple testing was applied, as implemented in the PMCMRplus R package [33]. The survival analysis, as well as the corresponding visualisation was carried out with the R packages “survival” [34] and “survminer” [35]. The survival times of animals that died due to dSCC were compared via multiple regression.

The significance levels were defined as follows: *p* ≤ 0.05 (weakly significant); *p* ≤ 0.01 (significant); and *p* ≤ 0.001 (strongly significant).

## 3. Results

### 3.1. Epidemiology

Cohort 1: Between 2013 and 2022, histopathological samples (tumours and non-neoplastic samples) from 1836 GSs, 2113 SSs, and 1595 MSs were submitted to the LABOKLIN pathology department. A neoplasm was diagnosed in 71.3% of the GS samples (*n* = 1309), in 72% of the SS samples (*n* = 1519), and in 65.7% of the MS samples (*n* = 1048). 

In total, 388 dSCCs (219 GSs, 153 SSs, and 16 MSs) were included in this study. The frequency of digital squamous cell carcinomas in the entire LABOKLIN material (tumours and non-neoplastic samples) was 11.9% in GS, 7.2% in SS, and 1% in MS. Related to the neoplastic samples from the LABOKLIN database, dSCCs were most common in GSs (219 dSCCs from 1309 neoplasms, 16.4%), followed by SSs (153 dSCCs from 1519 neoplasms, 10%), and MS (16 dSCC from 1048 neoplasms, 1.5%).

The gender distribution in the entire LABOKLIN sample collection was as follows: the GSs: 579 m, 240 mn, 483 f, 411 fn, and 123 unknown; the SSs: 691 m, 284 mn, 573 f, 360 fn, and 205 unknown; and the MSs: 506 m, 159 mn, 480 f, 314 fn, and 136 unknown. Significantly more samples were submitted from female (f/fn) giant schnauzers (*p* = 0.012) and miniature schnauzers (*p* < 0.001) than from males (m/mn). There was no obvious gender bias in the material from standard schnauzers in the LABOKLIN database.

Cohort 2: Additionally, 61 further dogs suffering from digital SCC (22 GS, 39 SS) from other veterinary pathology laboratories were included into the present study. This was performed because the clinical data of the outcomes were available from these animals from another study. Details about the signalment of these dogs are given in Supplementary Table S1.

Thus, a total of 478 dSCCs from 417 dogs (227 GSs, 174 SSs, and 16 MSs) were further analysed in this study. The coat colour, age, and sex of the animals are listed in Table 1, as far as this information was provided. Due to the small number of dogs with a pepper and salt coat, and due to the high number of dogs with an unknown coat colour, the coat colour was not included in the further statistical evaluation.

As far as the information about the sex was available from the entire 2013–2022 LABOKLIN database (Cohort 1), 52.2% of the GSs, 48.9% of the SSs, and 54.4% of the MSs were female/female neutered. In contrast, only 45% of the GSs and SSs, as well as 37.5% of the MSs with digital SCC were f/fn. Taking the gender proportion of the LABOKLIN database as standard, a predisposition to dSCC was identified for male GSs (*p* < 0.05). In SSs, the gender differences did not reach the level of significance. The group of MSs with dSCC was too small, and a statistical significance of sex predisposition could not be detected.

**Figure 1:**
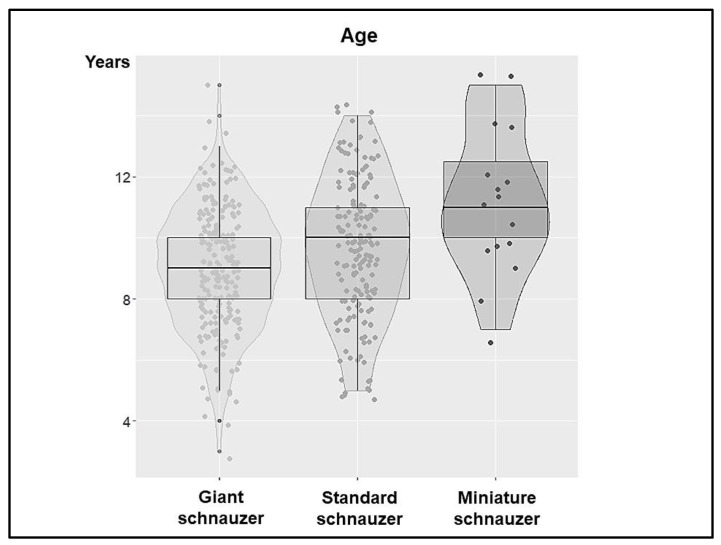
Age at initial diagnosis of digital squamous cell carcinoma in giant, standard, and miniature schnauzers. The violin plot shows the age distribution and frequency for the three dog breed variants. In addition, this distribution is indicated by the boxplot. Overall, there was a difference in age across all the breed variants (*p* < 0.001). There were strong significant age differences between the GSs and SSs (*p* < 0.001), as well as significant differences between the GSs and MSs (*p* = 0.001), whereas the age difference between the SSs and MSs was not significant.

The age of the dogs (Cohort 1 and 2) at the time of diagnosis was known for all 417 dogs and ranged from 3 to 15 years. The GSs were significantly younger than the SSs (*p* < 0.005) and MSs (*p* = 0.001) at the time of the dSCC diagnosis (Figure 1). 

### 3.2. Gross Findings of SCC

**Figure 2:**
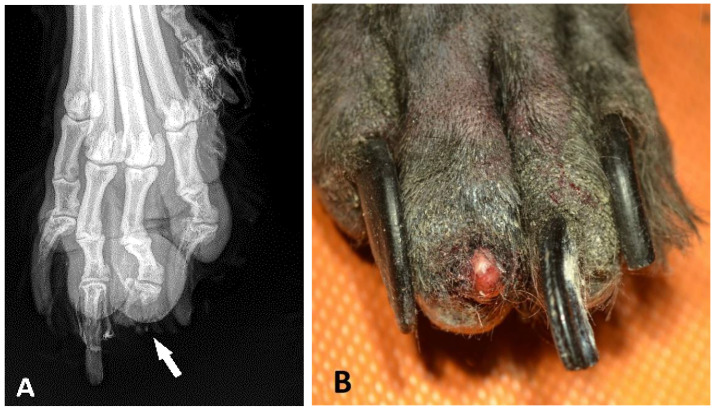
Paw of the left forelimb of a 10-year-old black male standard schnauzer. (**A**): On the dorsoventral radiograph of the distal phalanx of the third digit, a severe swelling of the soft tissue surrounding digit 3 is visible. The distal phalanx is missing and the bone had been resorbed, leaving the deformity on the digit (arrow). (**B**): The nail loss, redness, swelling, and oedema at the affected digit 3 of the paw from Figure (**A**).

In several cases, the submission forms did not contain detailed clinical information and it was difficult to obtain further data from the clients for various reasons. Where reported, the typical clinical history in all schnauzer breed variants was the number of nodular masses at the nail bed; the inflammatory thickening of the toe or nail bed; abnormal growth; deformation or loss of the nail; osteolysis; lameness; and/or unsuccessful antibiotic treatment. Osteolysis, which was identified by X-ray (Figure 2A), was reported on the submission form of 42 GSs, 45 SSs, and 3 MSs. In 79 cases, an ulcerative lesion of the digital skin was described. The nail of the digit was lost (Figure 2B) in 88 of 188 GSs (46.8%), in 53 of 138 SSs (38.4%), and in 4 of 12 MSs (33.3%). In 140 cases (59 GSs, 77 SSs, and 4 MS), the nail was not mentioned in the pathology report. However, there were no significant differences in the nail losses between the breed variants (*p* = 0.279).

Considering all 478 samples, the solitary distal phalanges/claws were submitted in 27 cases and the entire digit in 415 cases. In 32 cases, only biopsies were taken. In two dogs, the leg was submitted, and in two other cases, the material was not described in the pathology reports.

The gross appearance of the neoplastic toe did not vary significantly between the GSs and SSs: In the GSs, 106 nodular lesions (72.3%) and 34 cases of multinodular/diffuse swelling (23.3%) were described. In the SSs, 79 nodular lesions (73.8%) and 21 swollen toes (19.6%) were documented. In contrast, in the MSs, almost all digits showed nodular lesions (*n* = 10; 91%), and 1 swollen toe was described. In 13 cases (6 GS, 7 SS) in which the report stated that “no lesions” were found by macroscopy, a neoplastic destruction of the distal phalanx was histologically observed. In 214 cases (101 GSs, 108 SSs, and 5 MSs), the pathology reports did not state whether there were any obvious lesions during macroscopic preparation.

**Figure 3:**
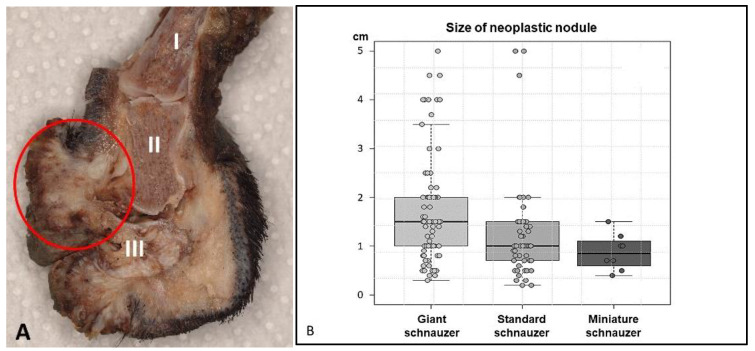
(**A**): The ulcerated mass (4.0 cm, circle) at the nail bed of digit 5 at the right hindlimb of a 7-year-old black giant schnauzer destructs the distal phalanx (III) (longitudinal cut of the amputated and formalin-fixed sample). (**B**): The boxplot shows significant differences of the size of nodular masses at the time of amputation of the digit with squamous cell carcinoma in giant, standard, and miniature schnauzers (*p* < 0.001). There were strong significant differences between the GSs and SSs (*p* < 0.001) and weakly significant differences between the GSs and MSs (*p* = 0.02), while there were no differences between the SSs and MSs (*p* = 0.659).

The 195 nodular neoplastic masses (Figure 3A) from 106 GSs, 79 SSs, and 10 MSs reached a diameter of up to 5.0 cm. The neoplastic nodules at the toes of the GSs were significantly larger (0.3–5.0 cm; median 1.5 cm) than those of the SSs (*p* < 0.001; 0.2–5.0 cm; median 1.0 cm) or MSs (*p* < 0.05; 0.4–1.5 cm; median 0.9 cm) (Figure 3B). 

**Figure 4:**
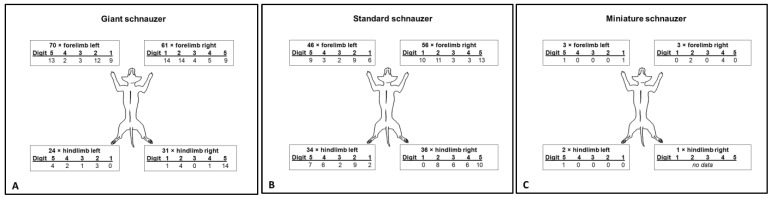
Number of legs and digits affected by squamous cell carcinoma in (**A**) giant, (**B**) standard, and (**C**) miniature schnauzers—as far as the data were available.

For most dogs, it was known which leg and toe were affected (Figure 4). In both the GSs and SSs, the forelimbs were significantly more often affected than the hindlimbs (GS 70%, *p* < 0.001; SS 59%, *p* = 0.022). In contrast, there was no significant difference between fore- and hindlimbs found in the MSs (*p* = 0.504). The right and left side of the body did not differ significantly in any group. Due to the low number of cases in the MSs, further statistical evaluation was only carried out for the SSs and GSs. 

**Figure 5:**
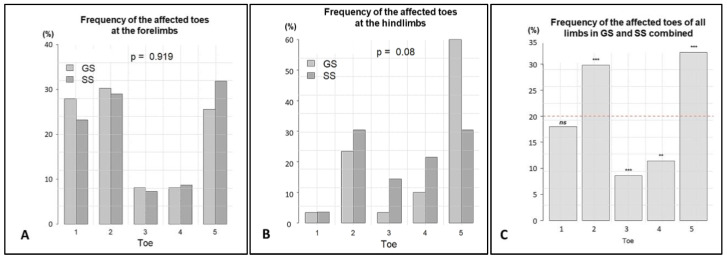
The relative frequencies of the affected toes were analysed. (**A**): At the forelimbs of giant (GSs) and standard (SSs) schnauzers, digits 3 and 4 were significantly less affected than digits 1, 2, and 5 (*p* < 0.001). No significant difference was found between the SSs and GSs (*p* = 0.919). (**B**): For the hindlimbs, there was an overall borderline association between the breed variants (GSs and SSs) and toes (*p* = 0.08). At the hindlimbs of the GSs, digit 5 was most commonly affected (*p* < 0.01). In the SSs, digit 1 was significantly less affected than the other toes of the hindlimbs (*p* < 0.01). (**C**): This figure shows the overall percentage of the affected toes combining the digits of all legs of the GSs and SSs. The dotted red line indicates the expected 20%. Only digit 1 showed no significant (ns) deviation from the 20%, while digits 2 and 5 were significantly (*** *p* < 0.001) more often affected, and digit 3 (*** *p* < 0.001) and digit 4 (**p < 0.01) were significantly less often affected.

In both breed variants (the GS and SS), digits 3 and 4 of the forelimbs were significantly less often affected than the other digits (both *p* < 0.001) (Figure 5A). Regarding the affected toes of the forelimbs, no significant difference was found between the SSs and GSs (*p* = 0.919).

For the toes of the hindlimbs (Figure 5B), there was a borderline association between the breed variants (GSs and SSs) and toes (*p* = 0.08). In the GSs, logistic regression for each toe yielded a significant difference for digit 5 of the hindlimbs (*p* < 0.01). This phenomenon was not observed in the hindlimbs of the SSs. On the contrary, in the SSs, digit 1 was significantly less often affected than the other toes of the hindlimbs (*p* < 0.01), while the differences between hindlimb digits 2, 3, 4, and 5 of the SSs were not significant.

If the toes of the fore- and hindlimbs of the GSs and SSs were combined (Figure 5C), there would be a 20% probability of dSCC occurring on each toe. However, only digit 1 corresponds to this probability, while digits 2 and 5 were significantly (*p* < 0.001) more often affected, and digit 3 (*p* < 0.001) and digit 4 (*p* < 0.01) were significantly less often affected.

### 3.3. Multiplicity

**Figure 6:**
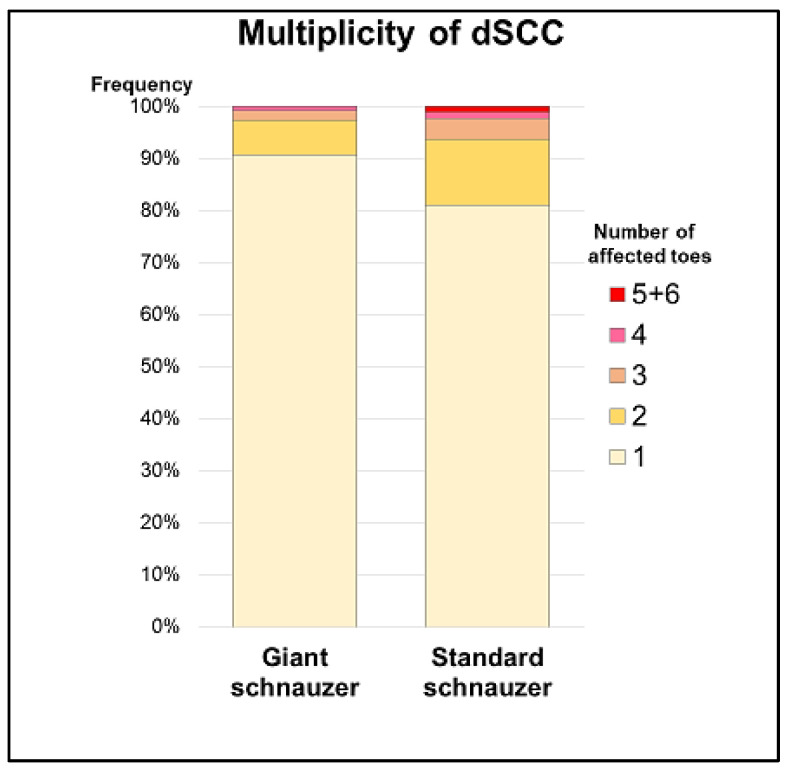
Multiple affected toes (>1) were significantly more common in standard than in giant schnauzers (*p* = 0.003).

**Figure 7:**
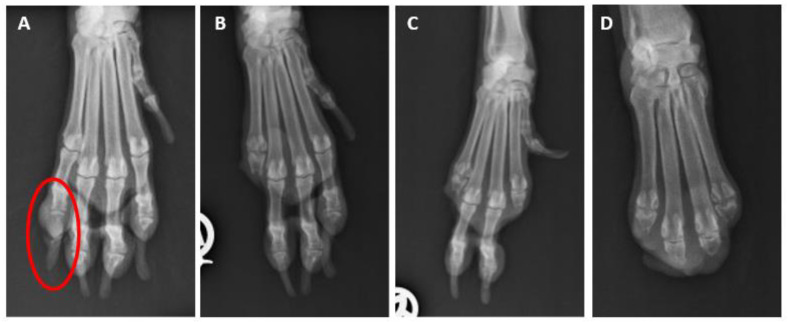
X-rays of the right front paw of a male standard schnauzer. (**A**): The osteolysis (circle) of the distal phalanx of digit 5 at the age of 9 years. (**B**): Status after amputation of the distal phalanx of digit 5 at the age of 9 years. (**C**): Status after the amputations of digits 2 and 5 at the age of 10 years. (**D**): Status after the amputation of all five digits at the age of 11 years. The © images were kindly provided by Dr. Andreas Haag, Mannheim, Germany.

In 54 dogs (21 GSs and 33 SSs), multiple dSCCs (2 to 6 affected toes) were reported. Multiplicity was significantly more common in the SSs (18.7%) than in the GSs (9.3%; *p* = 0.003; Figure 6). In our cohort, more than four affected digits were documented in the SSs alone (*n* = 4). When multiple dSCCs occurred, they were mostly located on different limbs but there were single cases with multiple affected toes on one leg (Figure 7).

For 46 dogs (18 GSs and 28 SSs) with multiple SCCs of the toes, the disease-free intervals were available. The disease-free intervals did not differ significantly between the GSs and SSs (*p* = 0.91). The time between the first and second dSCC ranged from 1 to 27 months in the GSs (median 13 months) and from 1 to 30 months in the SSs (median 12 months). A third toe was affected 1 to 25 months later (GS: *n* = 5, median 15 months; SS: *n* = 8, median 9 months). In one GS, a fourth toe developed digital SCC one month later. In four male black SSs, a fourth digit was affected 3 to 19 months later (median 7.5). Two of these dogs had dSCCs on a fifth digit 16 and 19 months later, respectively. One dog had lost six digits within 5 years due to dSCC and was euthanised at the age of 13 years because of a neoplastic event on another digit (not investigated).

### 3.4. Histology

**Figure 8:**
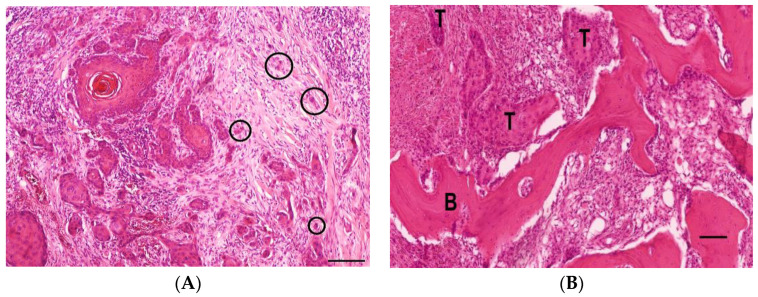
(**A**) Histology of a squamous cell carcinoma in a 6-year-old female giant schnauzer with intensive tumour cell budding (circles) at the invasive front (haematoxylin-eosin stain, bar = 100 µm). (**B**) Histology of a squamous cell carcinoma in a 13-year-old spayed female giant schnauzer with a squamous cell carcinoma (T) infiltrating the bone (B) (haematoxylin-eosin stain, bar = 100 µm).

In general, most toes showed epidermal ulceration and associated inflammation. Digital squamous cell carcinomas are characterised by the cords and islands of neoplastic epithelial cells with squamous differentiation, which infiltrate the dermis and are supported by moderate fibrovascular stroma. Tumour cell budding with detachments of tumour cell nests was common (Figure 8A). The neoplasms showed varying amounts of keratinization, cellular pleomorphism, as well as nuclear atypia and mitotic activity. Bone invasion and destruction was commonly seen (Figure 8B). The pathology reports did not indicate which phalanges were affected.

Surgical margins were free in 89.3% of the cases (196 GS, 158 SS, 12 MS). Infiltrated margins were mostly seen if only phalanx III was amputated (*n* = 27). In contrast, margins were infiltrated in only 6% of the 415 amputated digits (23 GSs, 18 SSs, and 3 MSs). There were no differences between the breed variants (*p* = 0.929). In 32 biopsies, margins could not be evaluated. In 36 cases, there was no information on resection margins in the pathology reports.

### 3.5. Clinical Outcome

The survival time after the pathological diagnosis of dSCC was available from 58 dogs (22 GSs and0 36 SSs). Furthermore, 7 dogs were still alive after an observation period of 1 to 4 years; 14 dogs died within the first year of diagnosis of dSCC; 13 dogs survived for more than 1 year but less than 2 years; and 24 dogs lived longer than 2 to 10 years. There were no differences in the survival times between the GSs and SSs (*p* = 0.209). No information was available if there was any treatment which accompanied the amputation.

The cause of death was given in 49 of the animals. Eight GSs and 13 SSs died from dSCC-related diseases. They were euthanized because of new SCCs on another toe (2×x GSs and 7× SSs), or they died from metastatic diseases from the dSCC (6× GSs and 4× SSs), or poor condition after digital amputation (2× SSs).

**Figure 9:**
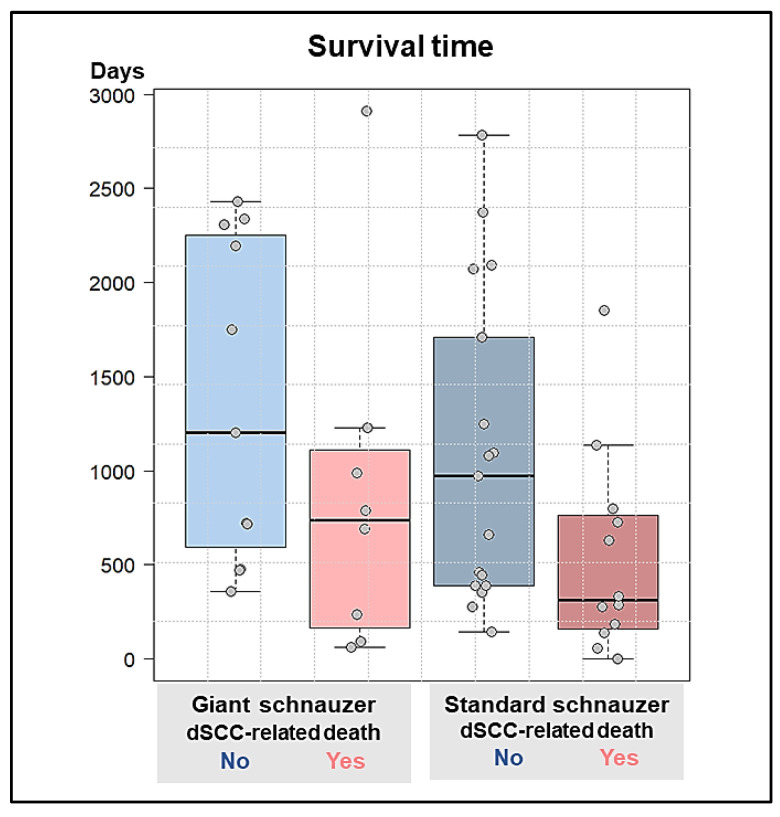
Boxplot of the survival time after diagnosis of the first digital squamous cell carcinoma: Multiple regression shows that both giant and standard schnauzers died significantly younger if digital squamous cell carcinoma was the cause of death (red) compared to those who died from other diseases (blue) (*p* = 0.004). There were no differences in the survival times found between giant and standard schnauzers (*p* = 0.209).

A total of 11 GSs and 17 SSs died from diseases that were not directly related to dSCC, such as other neoplasms (3× GSs and 4× SSs) or other general or cardiovascular diseases (8× GSs and 13× SSs). Both the GSs and SSs died significantly younger if digital squamous cell carcinoma was the cause of death compared to those who died from other diseases (*p* = 0.004) (Figure 9). 

Overall, the 1-year and 2-year survival rates were 70% and 50%, respectively. Dogs who died from SCC-related disease had a 50% rate of a 1-year survival and a 35% rate of a 2-year survival. Dogs who died from non-SCC-related diseases had a 1-year survival rate of 89.3% and a 2-year survival rate of 53%.

### 3.6. Metastases

In 59 dogs (22 GSs and 37 SSs), the status of their clinically detected organ metastases was available. In 47 cases (79.7%), the tumour was local. Metastases were clinically identified in 6 GSs and 6 SSs (20.3%). Metastases were found in the regional lymph nodes (*n* = 5), the proximal limb (*n* = 5), abdomen (*n* = 3), lungs (*n* = 2), and/or the pelvic region (*n* = 2). The dogs that developed metastases mostly had only one (*n* = 9) or two (*n* = 3) affected toes. In contrast, three or more digital SCCs had been sent in from 5 SSs; however, interestingly, no metastases were reported in these animals. Other parameters, such as clean margins, tumour site, or osteolysis, did not represent obvious clinical factors that could predict the risk of metastasis. Unfortunately, the size of the tumour masses was not given in the pathology reports from these 59 animals whose clinical course was known; thus, it was not possible to evaluate a correlation between the size and metastasis risk.

**Figure 10:**
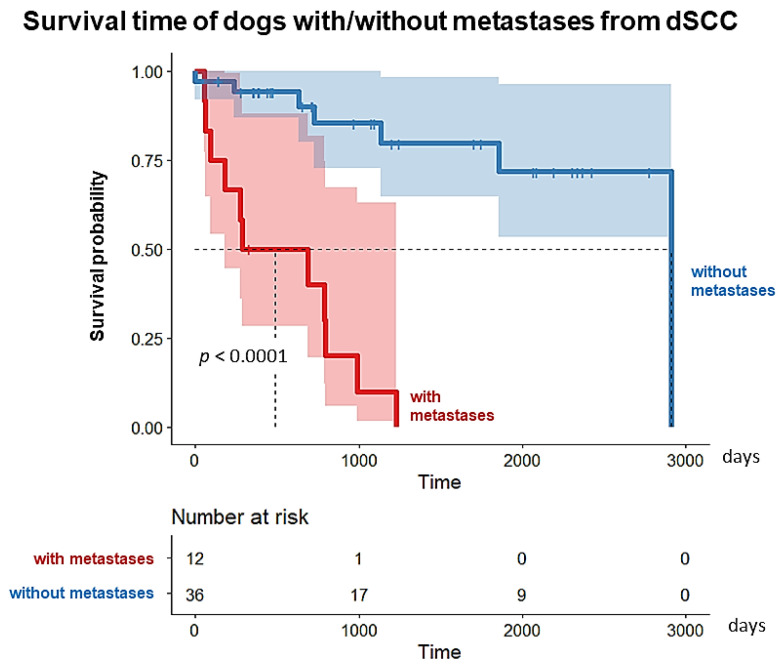
Survival analysis revealed a strongly significant difference in survival (*p* < 0.001) for schnauzer dogs with metastases (median survival: 489 days) compared to those without metastases (median survival: 2914 days) from which a survival time was given.

Cox regression revealed no significant differences between the breed variants of GS and SS (*p* = 0.169). Thus, survival time was considered independent of the variant: the survival time in dogs with metastases (*n* = 12) was significantly shorter (median 489 days) than in animals without metastases (*n* = 36; median 2914 days; *p* < 0.001; Figure 10).

## 4. Discussion

This study was the first to compare the clinical and macroscopic findings of squamous cell carcinoma on the digits of giant, standard, and miniature schnauzers. Black-coated dogs and large breeds, especially schnauzers, poodles, Labrador retrievers, and Rottweilers, are more predisposed to digital squamous cell carcinomas compared with other breeds [9,10,12,14,15,31]. However, it was noticeable that this tumour is rare in miniature schnauzers, while standard and giant schnauzers are commonly affected [8]. Interestingly, in the literature, the predisposing effect of size variants of the canine breeds has not been focussed on neoplastic diseases until now. However, it has also been reported that the short-legged terrier breeds (e.g., Jack Russel, West Highland White, Scottish terrier) are predisposed to urothelial carcinomas [36]. The genetic base of these effects needs to be investigated in further studies.

To relate our cohort to the schnauzer population, we used the data for tissue that was submitted for histopathological routine diagnostics between 2013 and 2022 for comparison. It was interesting to see that there was a similar number of samples in the database from each breed variant (1836 GSs, 2113 SSs, and 1595 MSs). The frequency of digital squamous cell carcinomas in the entire LABOKLIN material varied considerably among the breed variants: 11.9% in the GSs, 7.2% in the SSs, and 1% in the MSs. In another study, 23.7% of a total of 422 biopsy submissions from GSs and 10% of the 370 SS samples had digital SCC, but MSs were not mentioned [14]. However, there is one case report on a subungual pigmented SCC of digit 2 of the right hindlimb of an 11-year-old spayed female miniature schnauzer [37].

Of the 417 schnauzer dogs with digital SCC included in the present study, 54.4% were GSs, 41.7% were SSs, and 3.8% were MSs. In an older study of 38 digital SCCs in schnauzers, 12 GSs (32%), 24 SSs (63%), and 2 MSs (5%) were affected [31]. Why MSs are so greatly underrepresented in cases of digital SCC remains unclear. However, the predisposition to digital SCC in large-sized breeds in general has been described in various studies [12].

Dogs with dark coats are considered predisposed to dSCC compared with dogs with light coats [12,14,15]. In Germany, GSs and SSs are mainly black (puppies per year: 1 pepper and salt GS for every 10 black GS and about 1 pepper and salt SS for every 1.8 black SS). In contrast, the coat colour of MS puppies per year is as follows: 1 white to 1.3 black-silver to 3.4 black to 4.7 pepper and salt (https://psk-projekt.jimdo.com/unsere-rassen, accessed on 5 February 2023). As in another large retrospective study [14], a limitation of the present study was that for many dogs no information about the coat colour was available. Some of the dogs with black fur could be clearly identified based on the macroscopic descriptions in the pathology reports. Thus, analyses about the effects of the coat colour types in schnauzers with respect to dSCC will have to be conducted in further studies. Unfortunately, we had no information about the pedigrees, but familial clustering should also be taken into account [26].

About two-thirds of the male SSs and GSs with dSCC were intact. Looking at our database of all pathology samples from 2013–2022, a similar distribution is seen in the total population: 579 m/240 mn and 483 f/411 fn in GSs; 691 m/284 mn and 573 f/360 fn in SSs; and 506 m/159 mn and 480 f/314 fn in MSs. This generally indicates that intact schnauzers are more common in the population and there is probably no real predisposition. For further statistical evaluation, we did not differentiate between neutered and intact dogs because we did not have any information about the time of castration and thus the effect of neutering could not be interpreted. 

Data from the literature on gender predispositions to dSCC are contradictory: In two studies, male dogs (regardless of the breed variants) were more frequently affected than females [31,38,39]. Other authors did not confirm a sex predisposition but the breeds or their size variants were not taken into account [10,12,15,31]. Taking the gender proportion of schnauzers in the LABOKLIN database as standard, a predisposition to dSCC was identified for male GSs (*p* < 0.05). In contrast, the gender differences in SSs and MSs did not reach the level of significance. 

In the literature, the mean age for the onset of squamous cell carcinoma of the toe is given as 9 to 10 years, regardless of the breed [10,12,15,31,40]. In our study, we were able to show that standard and giant schnauzers are significantly younger than miniature schnauzers when they first develop squamous cell carcinoma of the toe. This would support the thesis that digital squamous cell carcinomas in MSs are spontaneous and are age-related tumours, whereas in SSs and GSs, the sum of different oncogenic factors may lead to dSCC at a significantly earlier age. The youngest GS in our cohort was 3 years old, the youngest SS was 5 years old. This is similar to other studies, except that the breeds and their size variants were not specified [15,31,40]. 

As described by most other authors, the forelimbs were more frequently involved than the hindlimbs [3,12,31,39]. However, there is one study of 21 cases of various breeds that did not confirm this [15]. There is one study [3] that described the distribution of the affected toes—regardless of the limb and the breeds: 15.1% on digit 1, 24.4% on digit 2, 24.4% on digit 3, 16.3% on digit 4, and 19.8% on digit 5. This is in contrast to the results of the present study of 115 toes from GSs and 125 toes from SSs, which showed that the incidence for SCC on digits 1, 2, and 5 of the forelimbs is high, while the inner digits 3 and 4 were significantly less affected in both breed variants. Some authors have speculated that the forelimbs may be more affected due to the higher mechanical stress when digging and running [12,39]. Yet, this cannot really explain why the outer toes are more often affected than the inner toes. At the hindlimbs, the affected toes varied between the SSs and GSs, but an explanation for this is still pending. 

A difficulty in veterinary practice is that the clinical picture of digital SCC, such as inflammation, lameness, and morphological changes, shows similarities to other neoplastic and inflammatory subungual diseases [9]. Radiological findings of lytic processes on the toes are not conclusive for neoplasms, especially for squamous cell carcinoma [11]. In a study of 117 dogs with masses on the toes, 83% of the 29 radiologically examined digital lesions that were accompanied by osteolysis also showed signs of malignancy, 17% had indicators of being both benign as well as showing inflammatory changes. About 80% of the squamous cell carcinomas had signs of osteolysis [3,11]. In the present study, osteolytic lesions were mentioned in the clinical report of the submission forms of only 45 GSs, 42 SSs, and 3 MSs. Nevertheless, these data were limited by the accuracy of the clinical history reports of this retrospective study.

Biopsy samples or fine needle aspiration may be helpful for making the final diagnosis [1,3]. However, the histopathological examination of the amputated toe provides an unambiguous diagnosis and gives information about histological aggressiveness, vascular invasion, and resection margins [1]. In the present study, 70.3% of 27 phalanx III amputations had infiltrated margins. In contrast, only in 6% of 415 entirely amputated digits were the margins infiltrated. There were no differences between the schnauzer breed variants. In general, dogs tolerated the amputation of a digit well [40]. Thus, as digital squamous cell carcinoma has malignant potential, high amputation is generally recommended to ensure that the edges of the incision are outside the tumour zone, including a safety margin.

Generally, deformation or loss of the nail are common findings in subungual SCC [31]. Nail loss was described in the pathology reports on the digits of 46.8% giant, 38.4% standard, and 33.3% miniature schnauzers, but these differences did not reach the level of statistical significance.

The gross appearance of the neoplastic tissue did not vary significantly between the GSs and SSs (about 73% nodular masses, 20% diffuse swelling). In contrast, in the MSs, almost all digits (91%) showed a nodular mass. There is no comparable data available in the literature.

Previous publications on digital SCC described nodular tumour masses of up to 6.0 cm (median 2.3 cm) without differentiating the breeds [3]. The neoplastic nodules at the toes of the GSs in our study were significantly larger than those of SSs (*p* < 0.001) or MSs (*p* < 0.05). About 30% of the masses in the GSs were ≥ 2.0 cm with the nail being lost, and they should have been clearly visible. The clinical reason for why the amputation was performed rather late remained unclear—especially in a breed which is well known to be predisposed. Unfortunately, the size of the tumour masses was not given for the animals of which the clinical course was known, such that a correlation between size and metastasis risk could not be evaluated in the present study and there are no comparable data available in the literature.

In the literature, the multiplicity of SCC (two to six affected toes) was reported in 5% to 23% of the cases in various breeds [3,10,12,14,31,31,31,41,42,42]. In a study of 106 dogs, the risk of developing digital SCC on an another toe within 2 years of initial diagnosis was 56% for giant schnauzers and standard poodles [14]. In our cohort, up to six toes with SCC were submitted from one dog over a time period of 6 years. Interestingly, in the present study, SSs (18.7%) were significantly more often affected by multiple digital SCCs than GSs (9.3%) (*p* = 0.003). However, we cannot exclude that there were not more cases with multiple digital SCCs that were not submitted to our laboratory. In cases of multiplicity, disease-free intervals of 2 years were commonly described in dogs of various breeds [12,14,26,43]. This period was similar (1–30 months) in the GSs and SSs in the present study. Another study of 79 dogs with digital SCCs, including 7 dogs with multiple affected digits, identified a shorter time of disease progression in the case of multiple compared with solitary events [3]. Unfortunately, there were not enough data about the survival time in dogs with multiple dSCCs to be evaluated in our study.

In general, digital SCC seem to have a relatively low propensity to metastasise (up to 23%) [3,10,12,14,39]. Radiologically, pulmonary metastases were found in 13% of the cases with squamous cell carcinoma, regardless of the breed [11]. Metastases can spread to various organs [3,42]. This is in line with the data from 59 dogs in the present study, which had a metastasis rate of 20.3% during an observation period of 3 months to 8 years after initial diagnosis. There were no apparent differences between the SSs and GSs. As described by other authors [3], dogs with metastases (6 GSs and 6 SSs) had a significantly shorter survival time in the present study. An interesting fact was that the animals with metastatic SCC predominantly had only one (*n* = 9) or two (*n* = 3) affected toes. Contrary to this, more than two digital SCCs had been observed in 5 SSs, but no metastases were reported in these animals. Further parameters, such as clean margins, tumour site, or osteolysis, are not obvious clinical factors that predict the risk of metastasis in the present study and other investigations [3,12].

A study of 79 dogs with digital SCC included 25 schnauzers (not further specified), which had a poorer outcome compared with other breeds [3]. This study showed that 24 dogs died from a progressive disease after 10–1468 days (median 302), while 31 died from tumour-unrelated causes 128–2370 days (median 745) after diagnosis; moreover, 24 dogs were still alive. Overall, the 1-year and 2-year survival rates were 81% and 60%, respectively [3]. In the present study, when including only GSs and SSs, the overall 1-year and 2-year survival rates were 70% and 50%, respectively. If only dogs who died from dSCC-related diseases are considered, the 1-year survival rate was 50% and the 2-year survival rate was 35%, which is similar to another study [39]. In the present study, no significantly different survival times were observed between the GSs and SSs. However, the small group size has to be taken into account. There was no information about how long the animals were observed without treatment or if there was additional treatment before or after amputation of the digit; this was such that the impact on the clinical outcome could not be evaluated in this study. Differences in survival times were identified depending on the origin of the canine digital SCC (subungual epithelium or other parts of the digital epidermis) [11]. The histology data of the digital squamous cell carcinomas were not evaluated in the present study but were previously described by other authors [1,12]. However, a grading system was established in another study by our group, in which giant, standard, and miniature schnauzers were not investigated separately [16]. It would be interesting to evaluate this in further studies, relating the histological grade to size variants of the schnauzer breeds, the multiplicity of the digital SCCs, as well as to the metastases and survival time.

In order to initiate appropriate treatment at an early stage, a regular check-up of all toes by means of adspection and palpation of the digits is recommended in standard and giant schnauzers. Taking into account the sole diagnosis by pathological findings and potential malignancy, rapid amputation should be recommended if anti-inflammatory treatment is unsuccessful as early surgery may prevent metastases [44]. We previously showed that the copy number (CN) variation at the KITLG gene significantly correlates with the likelihood of developing squamous cell carcinoma at the toe in black giant schnauzers [29]. In these dogs, this diagnostic test can help to estimate the individual risk of disease in black giant schnauzers and to sensitise pet owners accordingly if the CN value is high. An elevated value may encourage regular clinical examination of the dogs if necessary. Regular medical examination (adspection and palpation) of the toes, especially in such high-risk dogs, is therefore recommended.

In summary, for the first time, the clinical and macroscopic findings of squamous cell carcinoma at the digits of giant, standard, and miniature schnauzers were retrospectively analysed. Due to the inexplicably low number of cases from MS, statistical analyses were mostly limited to GSs and SSs. Although GSs and SSs are equally predisposed to squamous cell carcinoma of the toe, significant differences in the clinical picture between the two size variants were recognised in the parameters of sex, age, size of neoplastic nodules, multiplicity, and the affected toes of the hindlimbs. Regardless of the breed variants, the forelimbs were significantly more often affected, and the survival time in the case of metastases was significantly shortened. There were no differences between the SSs and GSs in the parameter gross findings, including nail loss, surgical margins, and overall survival time.

## 5. Conclusions

In conclusion, the results confirm that there are significant differences in the squamous cell carcinoma of the toe according to the size variant of the schnauzer breeds. In practice, regular adspection of the toes should be carried out in giant and standard schnauzers in order to detect clinical signs at an early stage, and early amputation of the entire digit is recommended to prevent recurrence and metastases. Multiplicity is a common event, especially in standard schnauzers, but is not obviously associated with metastases. 

## Figures and Tables

**Table 1:** The breed, coat colour, age, and sex of all dogs with digital squamous cell carcinoma included in this study (Cohorts 1 and 2).

Breed	Number of Samples	Coat Colour	Age (Years)	Sex
Giant schnauzer *n* = 227	*n* = 247	109 black, 118 unknown	3–15 median 9	82 m, 43 mn 44 f, 58 fn
Standard schnauzer *n* = 174	*n* = 215	88 black, 8 pepper and salt 78 unknown	5–14 median 10	71 m, 25 mn 39 f, 39 fn
Miniature schnauzer *n* = 16	*n* = 16	2 black, 1 black-silver, 1 pepper and salt, 12 unknown	7–15 median 11	8 m, 2 mn 1 f, 5 fn

Legend: m = male, mn = male neutered, f = female, and fn = female neutered.

## Data Availability

Data available only upon request due to privacy/ethical restrictions.

## Supplementary Materials

The following supporting information can be downloaded at: https://www.mdpi.com/article/10.3390/ani13121990/s1, Table S1: Cohort 2.
