# Supplementary material for: Comparative Study of Digital Squamous Cell Carcinoma in Giant, Standard, and Miniature Schnauzers
Source: Animals (Basel). 2023 Jun 14;13(12):1990. doi: 10.3390/ani13121990 (PMC10295616; doi:10.3390/ani13121990)
Supplement: Supplementary file 1 [file animals-13-01990-s001.zip › animals-2404691-supplementary.pdf]

Table S1: Cohort 2.

Cohort 2 Dog No.	breed GS= giant schnauzer SS=standard schnauzer,	coat colour	Age (years)	Sex	Number of affected toes	Number of affected legs
1	GS	black	6	fn	3	2
2	GS	black	10	mn	1	1
3	GS	black	12	m	1	1
4	GS	black	8	fn	1	1
5	GS	black	8	f	1	1
6	GS	black	7	f	1	1
7	GS	black	7	mn	1	1
8	GS	black	7	m	2	2
9	GS	black	10	m	1	1
10	GS	black	9	m	1	1
11	GS	black	7	f	2	2
12	GS	black	8	m	3	3
13	GS	black	7	m	1	1
14	GS	black	12	m	1	1
15	GS	black	10	f	1	1
16	GS	black	8	m	1	1
17	GS	black	6	m	1	1
18	GS	black	5	f	1	1
19	GS	black	9	m	1	1
20	GS	black	7	m	1	1
21	GS	black	4	m	1	1
22	GS	black	9	f	1	1
23	SS	black	8	m	1	1
24	SS	black	8	mn	1	1
25	SS	black	8	fn	1	1
26	SS	black	6	mn	3	2
27	SS	black	10	f	1	1
28	SS	black	8	fn	1	1
29	SS	black	8	m	1	1
30	SS	black	7	m	1	1
31	SS	black	10	f	1	1
32	SS	black	12	mn	2	2
33	SS	black	14	mn	1	1
34	SS	black	9	m	1	1
35	SS	black	11	m	2	2
36	SS	black	12	f	1	1
37	SS	black	10	m	1	1
38	SS	black	12	m	2	2
39	SS	black	10	mn	3	2

40	SS	black	6	m	1	1
41	SS	black	8	m	2	2
42	SS	black	7	f	2	2
43	SS	black	10	m	1	1
44	SS	black	7	m	6	3
45	SS	black	7	f	3	3
46	SS	black	10	fn	2	1
47	SS	black	13	mn	1	1
48	SS	black	10	m	3	3
49	SS	pepper-salt	10	fn	1	1
50	SS	pepper-salt	9	f	1	1
51	SS	pepper-salt	12	m	1	1
52	SS	pepper-salt	13	m	1	1
53	SS	pepper-salt	11	m	1	1
54	SS	black	9	f	1	1
55	SS	black	11	mn	2	2
56	SS	black	11	f	2	2
57	SS	black	11	m	1	1
58	SS	black	11	f	1	1
59	SS	black	14	m	1	1
60	SS	black	10	fn	1	1
61	SS	black	9	m	4	2
